# Children on dialysis as well as renal transplanted children report severely impaired health-related quality of life

**DOI:** 10.1007/s11136-018-1789-4

**Published:** 2018-01-27

**Authors:** Anouck Splinter, Lidwien A. Tjaden, Lotte Haverman, Brigitte Adams, Laure Collard, Karlien Cransberg, Maria van Dyck, Koen J. Van Hoeck, Bernd Hoppe, Linda Koster-Kamphuis, Marc R. Lilien, Ann Raes, Christina Taylan, Martha A. Grootenhuis, Jaap W. Groothoff

**Affiliations:** 1 Psychosocial Department, Emma Children’s Hospital, Amsterdam, The Netherlands; 2 Department of Pediatric Nephrology, Emma Children’s Hospital, Amsterdam, The Netherlands; 3 Department of Pediatric Nephrology, Hôpital Universitaire des Enfants-Reine Fabiola, Brussels, Belgium; 4 Department of Pediatric Nephrology, Centre Hospitalier Universitaire de Liège, Liege, Belgium; 5 Department of Pediatric Nephrology, Erasmus MC Sophia, Rotterdam, The Netherlands; 6 Department of Pediatric Nephrology, University Hospital Leuven, Leuven, Belgium; 7 Department of Pediatric Nephrology, University Hospital Antwerp, Antwerp, Belgium; 8 Department of Pediatrics, University Medical Centre, Bonn, Germany; 9 Department of Pediatric Nephrology, Radboud University Nijmegen Medical Centre, Nijmegen, The Netherlands; 10 Department of Pediatric Nephrology, University Medical Centre Utrecht, Utrecht, The Netherlands; 11 Department of Pediatric Nephrology, Ghent University Hospital, Ghent, Belgium; 12 Department of Pediatric Nephrology, University Hospital Cologne, Cologne, Germany

**Keywords:** Health-related quality of life, Renal replacement therapy, End-stage renal disease, Adolescents, Pediatrics

## Abstract

**Objectives:**

To assess health-related quality of life (HRQoL) across three renal replacement therapy modalities (preemptive transplant, non-preemptive transplant, and dialysis) in comparison with the healthy norm and other chronic health conditions, and to explore related patient factors.

**Study design:**

All prevalent end-stage renal disease (ESRD) patients aged 8–18 years who spent at least 6 months on their current treatment modality in the Netherlands, Belgium, and part of Germany were approached to complete the Pediatric Quality of Life Inventory 4.0 (PedsQL™) questionnaire. We determined the differences between groups on PedsQL™ mean scores, the proportion of children with an impaired HRQoL (≥ 1 SD lower than the healthy norm), the proportion of problems on individual items of the PedsQL™, and the effect of time on current treatment. Linear regression models were used to explore determinants of HRQoL.

**Results:**

192 out of 278 patients (20% preemptive transplant, 58% non-preemptive transplant, 22% dialysis) filled in the PedsQL™ (response rate 69%). Independent of treatment modality, patients had significantly lower mean scores and consequently higher proportions of impaired HRQoL on almost all domains compared to the healthy norm and other chronic health conditions. Patients with a preemptive transplant only reported higher scores on physical health compared to the other treatment modalities. Having comorbidities was the most important determinant associated with lower HRQoL scores.

**Conclusion:**

Dialysis and renal transplantation both have a severe impact on the HRQoL of children with ESRD. Physicians should be aware of this continuous burden. Furthermore, to develop tailored interventions for children with ESRD, qualitative studies are needed to gain more insight in the determinants of HRQoL in the different treatment modalities.

## Introduction

End-stage renal disease (ESRD) in children is a serious and life-threatening disorder. Renal transplantation is considered to be the optimal mode of renal replacement therapy (RRT) on all outcome aspects [1]. Successful transplantation most closely replicates the normal process of waste removal [2, 3]. In addition, transplantation attenuates the constraints imposed by maintenance dialysis therapy, such as severe dietary and fluid restriction and frequent hospital visits. Physicians therefore generally aim for preemptive transplantation, i.e., renal transplantation without receiving dialysis before [4]. Unfortunately, several technical, social, or immunological hurdles to transplantation may force physicians to initiate dialysis treatment [5].

Apart from the physical strain, the social implications of chronic RRT may even be more damaging in children. Refrainment of school attendance and participation in peer-related activities may severely compromise acquaintance of autonomy and academic skills [6, 7]. Assessment of health-related quality of life (HRQoL) may help to understand how children in different treatment modalities experience their illness and to identify potential factors of adverse experiences. Nevertheless, studies investigating HRQoL in children with ESRD are scarce, performed in relatively small numbers of patients and their results with respect to the effect of treatment modality are conflicting [8–12]. As a consequence, uncertainty exists to what extent renal transplantation indeed improves HRQoL in comparison to dialysis treatment in the pediatric ESRD population.

Therefore, we conducted a comprehensive study in a large cohort of children with ESRD that comprised all pediatric patients in the Netherlands, Belgium, and part of Germany. The aims were to assess HRQoL across three RRT modalities (preemptive transplant, non-preemptive transplant, and dialysis) in comparison with the healthy norm and other chronic health conditions, to examine the percentage of children with an impaired HRQoL, to explore specific problems, the effect of time on current treatment, and medical and psychosocial determinants.

## Methods

### Study design and procedures

This international HRQoL study was designed as a cross-sectional study within the Renal Insufficiency Therapy in Children: Quality Assessment and Improvement project (RICH-Q project) [13]. The RICH-Q database comprised medical and psychosocial characteristics of all pediatric ESRD patients from 2007 onwards in the Netherlands and Belgium and, from 2011, all children with ESRD treated in the center of Cologne, Germany. The HRQoL assessment was conducted utilizing the PedsQL™ Generic Core Scales 4.0 (PedsQL™) questionnaire. For this study, specific patients and the first completed questionnaire after a patient had spent at least 6 months on their current treatment modality was included in the current study. The inclusion criteria for patients in this study were patients with ESRD between 8 and 18 years who spent at least 6 months on their current treatment modality (preemptive transplantation, non-preemptive transplantation, or dialysis) between October 1, 2007 and December 31, 2014. Patients were excluded from the current study if they were younger than 8 years old and if they had spent < 6 months on their current treatment modality. Patients who met the inclusion criteria for this study, but either did not give informed consent or did not fill in the PedsQL™ are called ‘non-participants’.

We obtained ethical approval from the ethics boards of all 11 participating hospitals (Emma Children’s Hospital AMC (Amsterdam), Erasmus MC Sophia (Rotterdam), Radboud University Nijmegen Medical Centre, University Medical Centre Utrecht, Hôpital Universitaire des Enfants-Reine Fabiola (Brussels), Centre Hospitalier Universitaire de Liège, University Hospital Leuven, University Hospital Antwerp, Ghent University Hospital, University Medical Centre (Bonn), University Hospital Cologne).

### Clinical characteristics and socio-demographic data

Data on clinical characteristics were collected from the RICH-Q database: age, gender, mode of RRT, time in RICH-Q database, time on RRT, time on current treatment modality, the presence of comorbidities, and number of treatment changes. Comorbidity was defined as impairment in any organ other than the kidney or the involvement of a psychiatric condition.

Socio-demographic data of all participants and parents were collected at inclusion in the RICH-Q database using a questionnaire completed by one parent or caregiver on the following items: ethnic background (western vs. non-western), parental marital status, highest educational attainment of the parents, and country of residence. Educational attainment was categorized according to the highest successfully completed level of schooling: high level (high-level vocational training, “Hoger Beroeps Onderwijs,” “Fachhochschule,” or university) versus intermediate or low level. According to the definition of Statistics Netherlands [14], children were considered to be non-Western if they themselves or one or both parents had been born outside Western Europe.

### PedsQL™ Generic Core Scales 4.0

The PedsQL™ Generic Core Scales 4.0 is a 23-item, validated measure evaluating HRQoL of children by youth self-report and parent proxy-report. For this study only self-reports of children aged 8–18 years old were used. The questionnaire includes four subscale scores (physical, emotional, social, and school functioning) and yields a total HRQoL score and a psychosocial health summary score. The 23 items are answered on a 5-point Likert scale (1 = ‘‘never a problem’’ to 5 = ‘‘almost always a problem’’ over the past month) and reverse-transformed to a 0 to 100 metric, with higher scores representing better HRQoL. This measure has demonstrated good reliability and validity across pediatric populations, with internal consistency estimates ranging from .68 to .90 (Cronbach’s *α*) [15–17]. The reliability in our study ranged from * α* .79 to .92. Previous research provided Dutch data on a healthy norm and on other chronic health conditions [18].

### Statistical analysis

Baseline characteristics between the three treatment modalities were compared using Chi-square tests and independent *t* tests for categorical and continuous variables, respectively.

HRQoL outcomes were examined as follows. First, independent *t* tests were performed to compare the mean HRQoL scores between the three treatment modalities, with those in the healthy norm and with those in other chronic health conditions. Second, the percentages of patients with impaired HRQoL for all treatment modalities were compared with those of the healthy norm. Scores ≥ 1 SD below the mean of the healthy norm were considered as impaired. By definition, the prevalence of impaired HRQoL in the healthy norm is on average 16% for each domain. Chi-square tests were performed to compare the proportion of children impaired on HRQoL between the three treatment modalities and the healthy norm. Third, we studied the 23 individual items included in the PedsQL™ and calculated the proportion of patients who reported “often a problem” or “almost always a problem” on each item separately. Fourth, to assess the effect of time on current treatment, scores of patients who had received their current treatment for < 1 year were compared with those ≥ 1 year using independent *t* tests. Finally, multivariable linear regression analyses were performed to explore the association between HRQoL scores and potential determinants (age, gender, treatment changes, time on current treatment, treatment modality, presence of comorbidities, country of residence, marital status parents, education attainment, non-Western background). To include the appropriate determinants, univariate analyses were performed. Variables with *P* < .30 in one of the domains were included in the final regression analysis. Age and gender were included in all final regression analyses. Standardized regression coefficients (*β*) are reported, which express the strength of the association between the predictor variables and outcomes. For each regression model, the explained variance (*R*^2^) was determined, and it was tested using the F test. An effect size of explained variance of 0.02 was considered as small, 0.13 as medium, and 0.26 as large.

A significance level of 0.05 was used for all analyses. We used SPSS 22.0 for windows (SPSS Inc., Chicago, Illinois) for all statistical analysis.

## Results

Between October 2007 and December 2014, 278 children in the RICH-Q database met our inclusion criteria. Participants were divided into three groups based on their current treatment and treatment history: (1) patients with a preemptive renal transplant, i.e., without prior dialysis treatment, (2) patients with a non-preemptive renal transplant, i.e., after a period of time spent on dialysis, and (3) patients receiving dialysis (hemodialysis or peritoneal dialysis). Informed consent for HRQoL assessment was given by 254 children or their parents if children were < 12 years (91%). Of the 278 eligible children, 192 completed the PedsQL™ questionnaire (overall response rate 69%) after being 6 months on their current treatment. The 23 children and their parents who did not give their informed consent and the 63 non-responders together are denoted as non-participants (*N* = 86).

Medical and socio-demographic characteristics of the participants according to their treatment modality are presented in Table 1. The study sample included 38 (20%) children with a preemptive renal transplant, 112 (58%) children with a non-preemptive renal transplant, and 42 (22%) children receiving dialysis. Compared to the other two modalities, preemptively transplanted patients less often had comorbidities and had the highest proportion of parents with a high level of education. Patients on dialysis were older, more often from non-Western origin and had the lowest proportion of parents with a high-level educational degree.

**Table 1:** Medical and socio-demographic characteristics of participants according to treatment modality

	Preemptive transplant (*N* = 38)	Non-preemptive transplant (*N* = 112)	Dialysis (*N* = 42)	Healthy norm (*N* = 340)	Other chronic health conditions (*N* = 51)
Age at time of investigation^1,2,^*					
Mean (SD)	12.8 (3.1)	13.1 (3.6)	15.0 (2.9)	12.9 (2.3)	13.0 (2.2)
Median (range)^1,2,^*	13.0 (8.1–18.5)	13.7 (8.0–19.0)	15.9 (8.4–18.6)	12.8 (8.0–18.0)	13.0 (8.1–16.5)

	*N* (%)	*N* (%)	*N* (%)		
Gender F/M	17 (45)/21 (55)	55 (49)/57 (51)	20 (48)/22 (52)	102 (55)/84 (45)	29 (56)/22 (44)
Time in RICH-Q database (months)					
0–6	0 (0)	0 (0)	5 (12)		
6–11	20 (53)	13 (12)	15 (36)		
12–24	14 (37)	50 (44)	15 (36)		
> 24	4 (10)	49 (44)	7 (16)		
Time on RRT^2,3^					
6–11 months	19 (50)	2 (2)	17 (40)		
12–24 months	14 (37)	30 (27)	10 (24)		
> 24 months	5 (13)	80 (71)	15 (36)		
Time on current treatment modality (months)					
6–11	19 (50)	59 (53)	17 (40)		
12–24	14 (37)	35 (31)	10 (24)		
> 24	5 (13)	18 (16)	15 (36)		
Presence of comorbidities^1,3^	1 (3)	59 (53)			
Cardiovascular disease	0 (0)	7 (6)	18 (43)		
Neurological involvement^1,3^	0 (0)	17 (15)	4 (10)		
Gastro-intestinal disease	0 (0)	5 (5)	6 (14)		
Urogenital disorder	1 (3)	0 (0)	0 (0)		
Underlying syndrome	0 (0)	9 (8)	0 (0)		
Psychiatric condition	0 (0)	3 (3)	2 (5)		
Two or more treatment changes^1,3^	–	40 (36)	12 (29)		
Non- Western background^1,2,^*^,^^	6 (16)	31(28)	19 (45)	33 (18)	9 (17)
Parents married/ living together^α^	24 (80)	57 (74)	20 (74)		
High-level educational attainment parents^1,2,^*^,^,β^	13 (42)	22 (29)	2 (7)	84 (45)	23 (46)
Country of residence^1,2^					
The Netherlands	30 (79)	66 (59)	20 (48)		
Belgium	8 (21)	41 (37)	14 (33)	186 (100)	51 (100)
Germany	0 (0)	5 (4)	8 (19)		

^1^Significant difference between preemptive transplant and dialysis (*P* < .05)

^2^Significant difference between non-preemptive transplant and dialysis (*P* < .05)

^3^Significant difference between preemptive transplant and non-preemptive transplant (*P* < .05)

*Significant difference between dialysis and healthy norm and other chronic health conditions (*P* < .05)

^^^Significant difference between non-preemptive transplant and healthy norm (*P* < .05)

^α^Preemptive transplant: 21% missing data. Non-preemptive transplant: 24% data missing. Dialysis: 26% missing data

^β^Preemptive transplant: 18% missing data. Non-preemptive transplant: 32% missing data. Dialysis: 26% missing data

### Mean HRQoL scores

Mean scores (SD) for each PedsQL™ domain of patients, the healthy norm, and other chronic health conditions are presented in Table 2. No differences were found between the three treatment modalities and healthy norm as well as the three treatment modalities and other chronic health conditions on gender. Patients on dialysis were older, more often from non-Western origin and had a lower proportion of parents with a high-level educational degree than the healthy norm and other chronic health conditions. Compared to the healthy norm, non-preemptively transplanted patients were more often from non-Western origin and had a lower proportion of parents with a high-level educational degree.

**Table 2:** Mean HRQoL scores of children on RRT compared with healthy norm and other chronic health conditions

Subscale	Preemptive transplant			Non-preemptive transplant			Dialysis			Healthy norm			Other chronic health conditions^α^		
	*N*	Mean	SD	*N*	Mean	SD	*N*	Mean	SD	*N*	Mean	SD	*N*	Mean	SD
8–18 year old															
Total score	38	71.2**^,^^	17.8	112	68.5**^,^^^	16.7	42	69.1**^,^^	19.4	340	82.7	8.9	51	78.9	9.4
Physical health	38	78.6*^,1,2^	18.0	111	70.4**^,^^^	20.5	42	64.0**^,^^^	25.7	340	85.9	9.0	51	81.6	12.0
Emotional functioning	38	71.3	20.9	112	69.3**^,^^	20.9	42	70.8	21.5	340	77.1	14.3	51	75.2	15.3
Social functioning	38	75.7**^,^^	21.0	112	74.2**^,^^^	20.1	42	74.0**^,^^	21.1	340	88.1	12.0	51	83.3	12.8
School functioning	38	64.0**^,^^	19.3	107	63.2**^,^^^	18.2	40	65.5*	23.7	340	77.6	12.3	51	73.8	14.5
Psychosocial health	38	70.4**^,^^	17.5	112	68.5**^,^^^	16.8	42	69.8**^,^^	18.3	340	81.0	10.3	51	77.5	10.2

Independent sample *t* tests

^α^This group included patients with asthma (36%), congenital defect (14%), skin disease (6%), migraine (6%), and other diseases (38%)

*Versus healthy norm *P* < .05

**Versus healthy norm *P* < .001

^^^Versus other chronic health conditions *P* < .05

^^Other chronic health conditions *P* < .001

^1^Significant difference between preemptive transplant and dialysis

^2^Significant difference between preemptive transplant and non-preemptive transplant

Overall, patients reported significant lower scores compared to the healthy norm and other chronic health conditions. Regarding physical health, preemptively transplanted patients disclosed similar scores compared to the other chronic health conditions. Preemptively transplanted patients and patients on dialysis reported similar scores compared to the healthy norm on emotional functioning. Preemptively transplanted patients scored significantly higher than both non-preemptive transplant and dialysis patients on physical health. No statistically significant differences were found across the three treatment modalities in other domains.

### Proportion of patients with impaired HRQoL

**Fig. 1:**
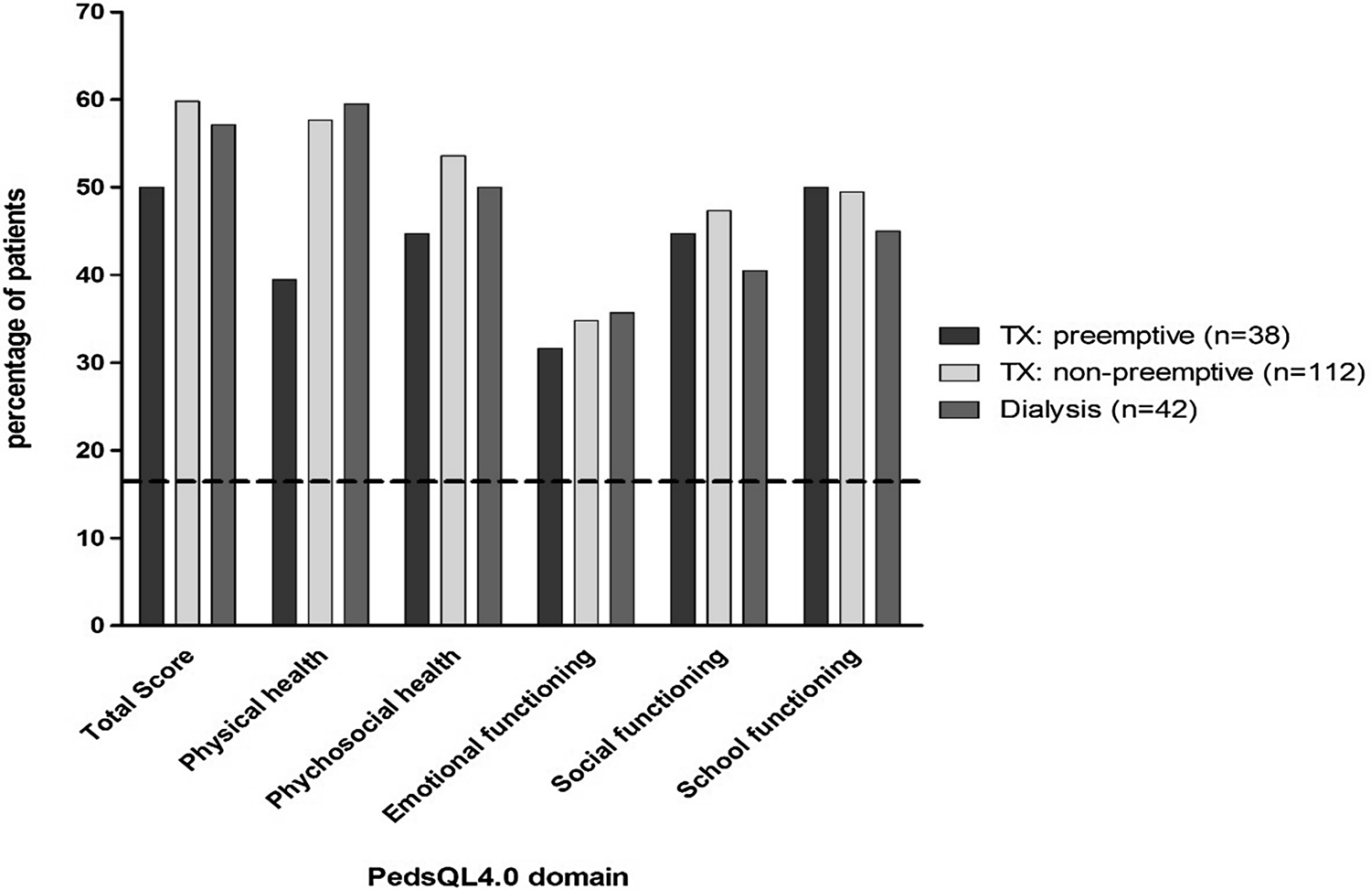
Proportion of children with impaired HRQoL (PedsQL™ score > 1 SD below the healthy norm). Dotted line represents the 16% of the Dutch healthy norm with impaired quality of life according to their PedsQL™ score. Patients in all three treatment modalities reported more often impaired HRQoL in all domains compared with the healthy norm (*P* < .05)

Impaired HRQoL was significantly more often found in all three treatment modalities compared to the healthy norm for all domains and was equally prevalent in all three treatment modalities (Fig. 1).

### Individual PedsQL™ items

The proportion of patients who reported “often a problem” or “almost always a problem” for individual PedsQL™ items is presented in Table 3 for each item separately. The items “I miss school to go to the doctor or hospital” and “I cannot do things that other kids my age can do” were the most reported problems in all three treatment modalities.

**Table 3:** Proportion of patients reporting “often a problem” or “almost always a problem” on the PedsQL™ subscales

Domain	Items	Preemptive transplant	Non-preemptive transplant	Dialysis	Healthy norm	Other chronic health conditions
		%	%	%	%	%
Physical health	It is hard for me to walk more than one block	8	12	17	0	0
	It is hard for me to run	8	20	25	2	6
	It is hard for me to do sports activity or exercise	17	15	29	2	8
	It is hard for me to lift something heavy	8	20	24	3	2
	It is hard for me to take a bath or shower by myself	8	6	26	0	0
	It is hard for me to do chores around the house	3	14	17	0	2
	I hurt or ache	5	7	12	5	18
	I have low energy	10	20	24	4	8
Emotional functioning	I feel afraid or scared	6	11	5	3	6
	I feel sad or blue	3	9	10	5	12
	I feel angry	21	16	20	3	8
	I have troubling sleeping	13	13	12	7	6
	I worry about what will happen to me	11	12	10	4	6
Social functioning	I have trouble getting along with other kids	11	7	2	1	4
	Other kids do not want to be my friend	6	3	7	3	2
	Other kids tease me	3	4	0	2	4
	I cannot do things that other kids my age can do	21	23	34	1	8
	It is hard for me to keep up when I play with other kids	8	14	19	1	0
School functioning	It is hard to pay attention in class	13	20	13	6	6
	I forget things	19	18	23	8	6
	I have trouble keeping up with my schoolwork	16	22	13	4	12
	I miss school because of not feeling well	5	12	12	1	6
	I miss school to go to the doctor or hospital	29	26	32	1	2

### Impact of time on current treatment on HRQoL

Group differences between the three treatment modalities on mean scores (SD) from patients who received their current treatment for < 1 year compared to scores from patients who had been on the same treatment ≥ 1 year were assessed (Table 4). Similar scores in all domains were found among dialysis and preemptively transplanted patients. Non-preemptively transplanted patients who had received their graft ≥ 1 year ago reported significantly lower scores compared to patients who had been transplanted < 1 year ago on the total score, social functioning, school functioning, and psychosocial health domain scores. Compared to the healthy norm, all three treatment modalities had significantly lower total HRQoL scores regardless of they had spent less or more than 1 year on their current treatment.

**Table 4:** Mean HRQoL scores of children < 1 year compared with children ≥ 1 year on their current treatment modality

Subscale	Preemptive transplant				Non-preemptive transplant				Dialysis				All treatment groups				Healthy norm		Other chronic health conditions	
	< 1 year		≥ 1 year		< 1 year		≥ 1 year		< 1 year		≥ 1 year		< 1 year		> 1 year					
	*N*	Mean (SD)	*N*	Mean (SD)	*N*	Mean (SD)	*N*	Mean (SD)	*N*	Mean (SD)	*N*	Mean (SD)	*N*	Mean (SD)	*N*	Mean (SD)	*N*	Mean (SD)	*N*	Mean (SD)
Total score	19	72.8 (18.2)*	19	69.5 (17.6)*^,^^	59	72.6 (16.1)**^,^^	53	64.0 (16.4)^1,^**^,^^^	22	64.9 (19.1)**^,^^	20	73.6 (19.1)*	100	71.0 (17.3)**^,^^^	92	67.2 (17.6)**^,^^^	340	82.7 (8.9)	51	78.9 (9.4)
Physical health	19	77.0 (18.4)*	19	80.2 (18.0)	59	70.8 (21.4)**^,^^	52	70.0 (19.6)**^,^^^	22	58.4 (26.7)**^,^^	20	70.2 (23.8)*	100	69.3 (22.8)**^,^^^	91	72.2 (20.5)**^,^^	340	85.9 (9.0)	51	81.6 (12.0)
Emotional functioning	19	73.7 (20.3)	19	69.0 (21.8)	59	71.8 (21.6)	53	66.6 (20.0)**^,^^	22	66.1 (23.0)*	20	75.9 (19.0)	100	70.9 (21.6)*	92	69.1 (20.3)*^	340	77.1 (14.3)	51	75.2 (15.3)
Social functioning	19	75.5 (21.4)*	19	75.8 (21.1)*	59	78.6 (17.8)**	53	69.3 (21.4)^1^**^,^^^	22	71.4 (21.9)*^,^^	20	76.9 (20.4)*	100	76.5 (19.5)**^,^^	92	72.3 (21.2)**^,^^^	340	88.1 (12.0)	51	83.3 (12.8)
School functioning	19	64.5 (18.5)*	19	63.4 (20.6)*^,^^	58	67.3 (17.9)**^,^^	49	58.4 (17.5)^1^**^,^^^	20	63.7 (23.3)*	20	67.3 (24.5)	97	66.0 (19.1)**^,^^	88	61.5 (20.0)**^,^^^	340	77.6 (12.3)	51	73.8 (14.5)
Psychosocial health	19	71.2 (17.7)*	19	69.5 (17.6)*	59	72.5 (16.1)**	53	64.0 (16.4)^1^**^,^^^	22	66.6 (17.1)*^,^^	20	73.4 (19.4)	100	71.0 (17.3)**^,^^	92	67.1 (17.6)**^,^^^	340	80.9 (10.3)	51	77.5 (10.2)

Independent sample *t* tests

^1^Significant difference between > 1 and < 1 year on treatment

*Versus healthy norm *P* < .05

**Versus healthy norm *P* < .001

^^^Versus other chronic health conditions *P* < .05

^^Other chronic health conditions *P* < .001

### Determinants of HRQoL

The presence of comorbidities was the only determinant associated with lower scores in almost all HRQoL domains, except for school functioning. Furthermore, having single parents was associated with lower HRQoL scores on emotional functioning.

## Discussion

In this international multicenter study covering all centers in the Netherlands, Belgium, and a small part of Germany, impaired HRQoL was found in children with ESRD. Except for higher scores on physical health in patients with a preemptive transplant, similar HRQoL scores across all treatment modalities were reported. All patients reported problems with regard to social functioning (e.g., restriction in activities) and school functioning (missing school due to hospital visits). Presence of comorbidities was the most important determinant for lower HRQoL scores.

In line with previous reports, this study found significant lower PedsQL™ scores among children with ESRD compared to the healthy norm as well as compared to other chronic health conditions in almost all domains [8, 9, 11, 12]. Given the broad consensus among health professionals and patients regarding the positive effect of a functioning transplant in the ESRD population, the hypothesis was that transplanted patients would report a higher HRQoL compared to patients on dialysis. Indeed, patients with a preemptive transplant reported higher scores on the domain of physical health compared to patients in the other treatment modalities, but we found no differences in other HRQoL domains across the treatment modalities. This contrasts with findings from adult ESRD studies which showed higher scores in transplanted compared to dialysis patients, especially in the domains of general health perceptions, social functioning, and bodily pain [19, 20], but is in accordance with adults who had their onset of RRT in childhood [21].

Regarding specific HRQoL domains, we found important decrements in terms of social and school functioning in both dialysis and transplanted children. Up to 26% of non-preemptively and 29% of preemptively transplanted patients reported problems to go to school because of hospital visits. Fear of losing the graft, overprotection of families, and the need for frequent clinical check-ups may have led to these results [22]. With respect to impaired social functioning, patients receiving a preemptive transplant have not experienced the benefit of transplantation compared to the dialysis status and may therefore suffer more from the adverse effects of transplantation, such as side effects of immunosuppressive therapy, in particular weight gain, gingival hypertrophy, acne, and Cushingoid appearance. These are important but too often underestimated problems in transplanted patients, particularly for adolescents which could have an important impact on their perceived quality of life [23]. In addition, patients on dialysis may have high expectations of the benefits of receiving a transplant, but may also realize after some time that transplantation is not a cure and goes on with its own burden. Our finding that non-preemptively transplanted patients who had received their graft ≥ 1 year ago reported worse scores on several domains compared to patients who had been transplanted < 1 year ago supports this hypothesis. In dialysis patients we found an opposite trend: patients who had received dialysis treatment for ≥ 1 year reported better (non-significant) HRQoL compared to patients who were on dialysis treatment for less than a year. This may imply that children on dialysis adjust to their situation over time and partly learn to cope with their disease and treatment. This change in health status is called ‘response shift’ and refers to a change in the meaning of one’s self-evaluation of a target construct [24]. This change in health status could be the result from changes in internal standards, values, and redefinition of the target construct. Future research should focus on response shift in the pediatric ESRD population. Furthermore, a disease-specific assessment instrument could shed light on more specific issues that patients with ESRD are facing. The PedsQL™ 3.0 ESRD module was developed, using the PedsQL methodology, to obtain detailed information about what may impact the HRQoL of pediatric patients with ESRD [25]. This instrument contains seven domains: general fatigue, about my kidney disease, treatment problems, family and peer interaction, worry, perceived physical appearance, and communication. A combination of both the generic and disease-specific module of the PedsQL™ is recommended to gain insight in the HRQoL of patients with ESRD [26].

In accordance with previous studies [27], the existence of comorbidities was the most important determinant of lower scores on almost all HRQoL domains. Except for having single parents on emotional functioning, we found no other socio-demographic determinants to be associated with lower HRQoL scores. Since we have specified different comorbidities but only included the existence of comorbidity in the regression analysis, a comorbidity index for pediatric patients should be developed to assign different weights to each comorbidity [28]. Furthermore, the literature regarding determinants of HRQoL among children with ESRD is inconsistent, especially with regard to emotional functioning. Previous studies found that female gender, low parental educational attainment, and non-Western background were associated with lower HRQoL scores [9, 14, 29]. Small sample sizes and variable measures of HRQoL may contribute to this divergence of identified determinants in the pediatric ESRD population. In addition, it is possible that studies do not include determinants specifically important to HRQoL such as strategies of coping with the disease, social support, and psychological problems [30]. Such factors should be studied in more detail in the future.

Current results suggest that emphasis should be put on informing patients about all possible consequences and complications of a renal transplantation. This might help patients to equip and prepare, and might also avoid unrealistic high expectations of life after receiving the transplant. In addition, studies have suggested that valid information is vital for promoting adherence to medication regimens and maintaining graft survival [31, 32]. One of the most consisting findings was the frustration among children in all treatment modalities of not being able to keep up with peers as well as to equally participate in social activities and school.

Physicians should be aware of the continuous burden with respect to social development and school performance, even after transplantation, and try to adjust their practice where possible in order to allow them to live life as normal as possible.

### Limitations

Some limitations of the study should be emphasized. First, the participants in this study do not cover the entire pediatric RRT or the entire group of patients in the RICH-Q database. We were therefore not able to perform any comparisons between participants and non-participants at the moment of our HRQOL assessment. Nevertheless, to provide information about potential differences between the three treatment modalities we compared baseline characteristics, i.e., at time of inclusion in RICH-Q database. Second, data of healthy children and children with other chronic health conditions were collected in the Netherlands. It is questionable whether these data can be used as a reference group for patients from Belgium and Germany. However, according to our regression analysis, country was not associated with HRQoL scores. Third, the relatively small dialysis and preemptively transplanted sample size requires us to interpret current HRQoL comparisons with caution. Furthermore, children on dialysis were older compared to the healthy norm and other chronic health conditions but age was not associated with HRQoL scores. Finally, a generic HRQoL tool was used in order to compare children with ESRD with healthy children and children with other chronic health conditions. Possibly, the addition of the PedsQL 3.0 ESRD module would have captured more disease-specific aspects of HRQoL functioning in the different treatment modalities.

## Conclusions

Chronic dialysis and renal transplantation both could have a severe impact on the psychosocial development of children, especially on their social development and academic performance. Even preemptive transplantation, although it is the most favorable option for children with ESRD in physical terms, can have an important impact on their HRQoL. Physicians should be aware of this and adjust their practice where possible in order to improve autonomy development of these patients. Furthermore, to develop tailored interventions for children with ESRD, qualitative studies are needed to gain more insight in the determinants of HRQoL.
